# Recent progress of low-temperature plasma technology in biorefining process

**DOI:** 10.1186/s40580-023-00386-2

**Published:** 2023-08-24

**Authors:** Lusha Qin, Oi Lun Li

**Affiliations:** 1 School of Food Science, Henan Institute of Science and Technology, Henan 453003 Xinxiang, People’s Republic of China; 2 School of Materials Science and Engineering, Pusan National University, 30 Jangjeon-dong, Geumjeong-gu, Busan, 46241 South Korea

**Keywords:** Low-temperature plasma, Biomass pretreatment, Biomass gasification, Biomass liquefaction, Chemo-catalysis

## Abstract

In recent years, low-temperature plasma-assisted processes, featuring high reaction efficiency and wide application scope, have emerged as a promising alternative to conventional methods for biomass valorization. It is well established that charged species, chemically energetic molecules and radicals, and highly active photons playing key roles during processing. This review presents the major applications of low-temperature plasma for biomass conversion in terms of (i) pretreatment of biomass, (ii) chemo fractionation of biomass into value-added chemicals, and (iii) synthesis of heterogeneous catalyst for further chemo-catalytic conversion. The pretreatment of biomass is the first and foremost step for biomass upgrading to facilitate raw biomass transformation, which reduces the crystallinity, purification, and delignification. The chemo-catalytic conversion of biomass involves primary reactions to various kinds of target products, such as hydrolysis, hydrogenation, retro-aldol condensation and so on. Finally, recent researches on plasma-assisted chemo-catalysis as well as heterogeneous catalysts fabricated *via* low-temperature plasma at relatively mild condition were introduced. These catalysts were reported with comparable performance for biomass conversion to other state-of-the-art catalysts prepared using conventional methods.

**Graphical Abstract:**

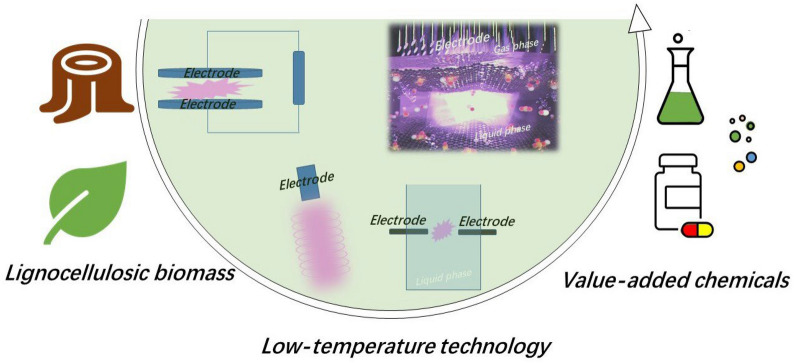

## Introduction

The depletion of traditional fuels and related environmental problems have made the development of renewable and clean energy a current focus of research [1, 2]. It is widely acknowledged that biomass energy is one of the most promising alternatives based on its great abundance and low negative effect on environment [3–5]. In terms of chemical composition, lignocellulosic biomass waste is primarily composed of hemicellulose, cellulose, lignin, and some extractives and ash [6]. Except for these three major components, the direct derivatives and some upstream products are typically generated by pretreatment processing and delignification [7]. The polysaccharides (hemicellulose and cellulose) and lignin can be converted into key platform chemicals such as monosaccharides, lignin monomers, and a variety of other value-added products [8–10]. For example, sugar alcohols such as sorbitol, mannitol, and xylitol are important chemicals in medicine, cosmetics, and food industry [11]. In addition, biomass-derived ethane, propane, and hexane are regarded as essential components of gas fuels [12]. Lignin is another component of biomass feedstock. Specifically, owing to their abundance of aromatic rings and methoxy, phenolic hydroxy, alkyl, and fatty alcohol groups, some pure, value-added, and commodity chemicals can be produced by selective functionalization. For instance, the lignin-derived monomers, such as phenol, guaiacol, and syringol are significant chemical to produce dyes, drugs, phenolic resins, adhesives, and so on [13–15].

To economically realize environmental-friendly biorefineries, various catalytic approaches (e.g., microbial fermentation, pyrolysis, and chemo-catalysis) have been explored in recent years [16]. However, although the enzyme shows comparable productivity to that of other processes, the high cost makes the microbial fermentation for converting lignocellulosic biomass is a great impediment in large scale [17]. Pyrolysis is commonly utilized to produce downstream chemicals at high temperatures, it suffers from the issues of intensive reaction conditions and low selectivity of the target products. The transformation of lignocellulosic biomass via chemo-catalysis process over the homogeneous or heterogeneous catalyst has made great achievement in past decades. Recently, an increasing research trend is focus on the development of active and recyclable catalyst for sustainable development [11].

**Scheme 1:**
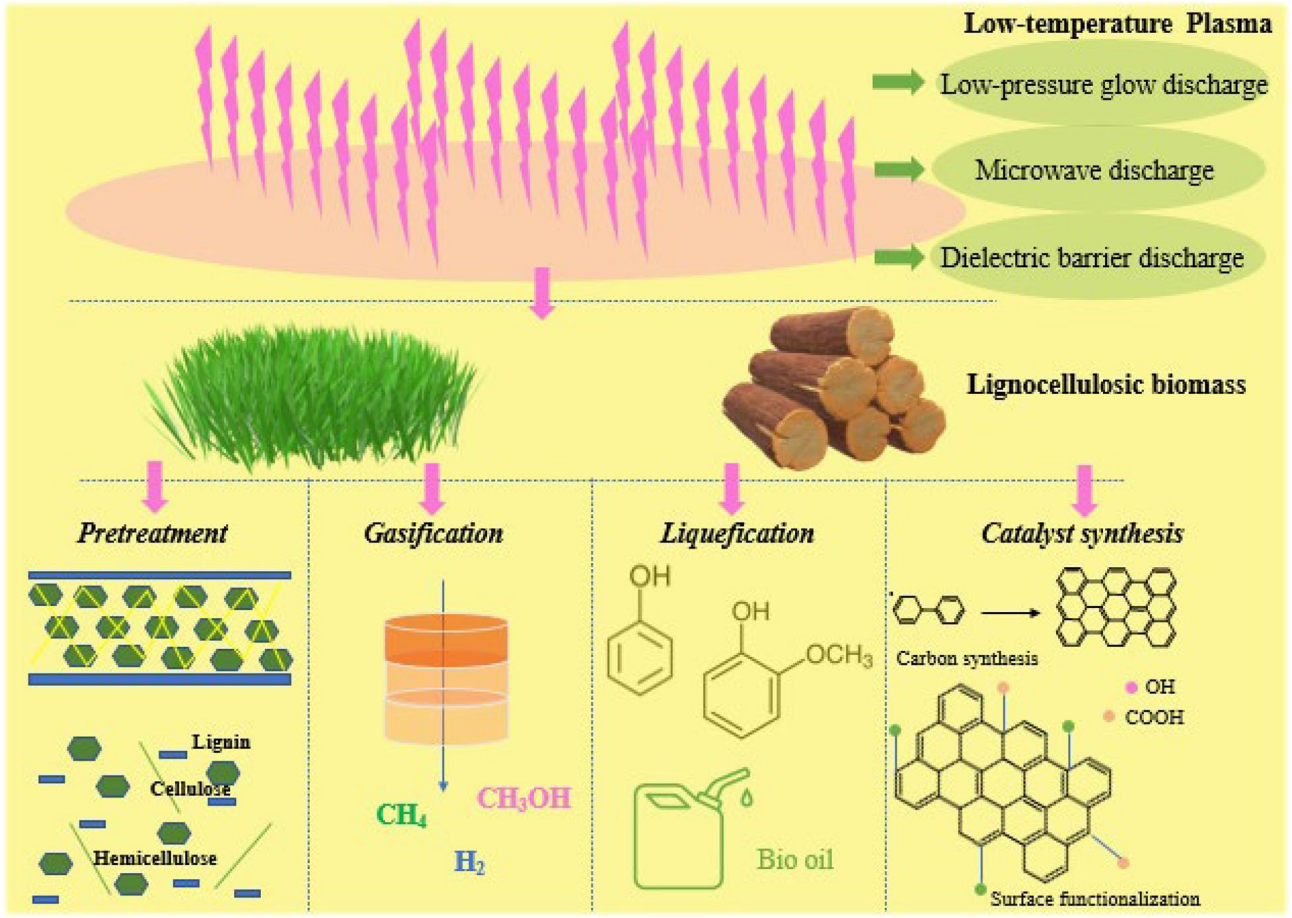
Schematic illustration of low-temperature plasma on biomass conversion

Low-temperature plasma has gained much research interest for biomass conversion over the past few decades since its advantages of low cost, high efficiency, and low waste generation [18–21]. Among the state-of-the-art technologies, plasma process enables the pretreatment of raw biomass, gasification of biomass to syngas and hydrogen, liquefaction of raw biomass or biomass-derived feedstock, and synthesis of heterogeneous catalysts for further chemo-catalytic conversion, as depicted in Scheme 1 [22, 23].

Overall, this review provides basic knowledge at the interdisciplinary research between plasma engineering and biomass conversion. Herein, we review the application of various plasma technologies for lignocellulosic biomass upgrading, aiming to provide a scientific study and investigate technical challenges of this emerging technology.

## Fundamental of low-temperature plasma and reactor designs

In theory, plasma is an ionized gas that presents a highly energetic state. Plasma technology can be classified as thermal (equilibrium plasma) or non-thermal (non-equilibrium plasma) plasma according to the energy level, temperature, and electronic density [24, 25]. But thermal plasma has the drawback of extremely high processing temperature. The over-transformation and the generation of some unintentionally undesired products might occur during thermal plasma processing. The post-quenching and electrode corrosion lead to the complex operation and limited energy efficiency.

Non-thermal plasma (non-equilibrium plasma) is considered as low-temperature plasma due to its unique properties. While the electrons are highly excited within the plasma gas, other components such as ions and gas molecules remain relatively stable. In specific, the electrical energy is directed to plasma to merely energize electrons while most of the ions and neutral substances (gas molecules) remain at room temperature [26]. Also, the generation of non-thermal plasma requires significantly less energy compared to thermal plasma, and it can be carried out at or slightly below atmospheric pressure. During the plasma discharge process, a great number of electrons, ions, and other species are accelerated to acquire kinetic energy, followed by many reactive chemical species generated from such induced ionization, excitation, and dissociation reaction.

Therefore, a collection of co-existing chemically reactive species, electrons, the charged particles, as well as electromagnetic waves play as the key factors in gas plasma for biomass processing. In addition, integrating plasma processing with the chemo-catalytic process can enhance the reaction efficiency for biomass pretreatment, gasification, liquefaction, and catalyst synthesis. Initially, the plasma technology was developed greatly for the biomass gasification, which is mainly operated in gas environment [27]. In recent years, plasma sustained directly in liquids or interacting with liquids are being intensively investigated to address fundamental properties, especially for the liquefaction of biomass into value-added chemicals [28].

**Fig. 1:**
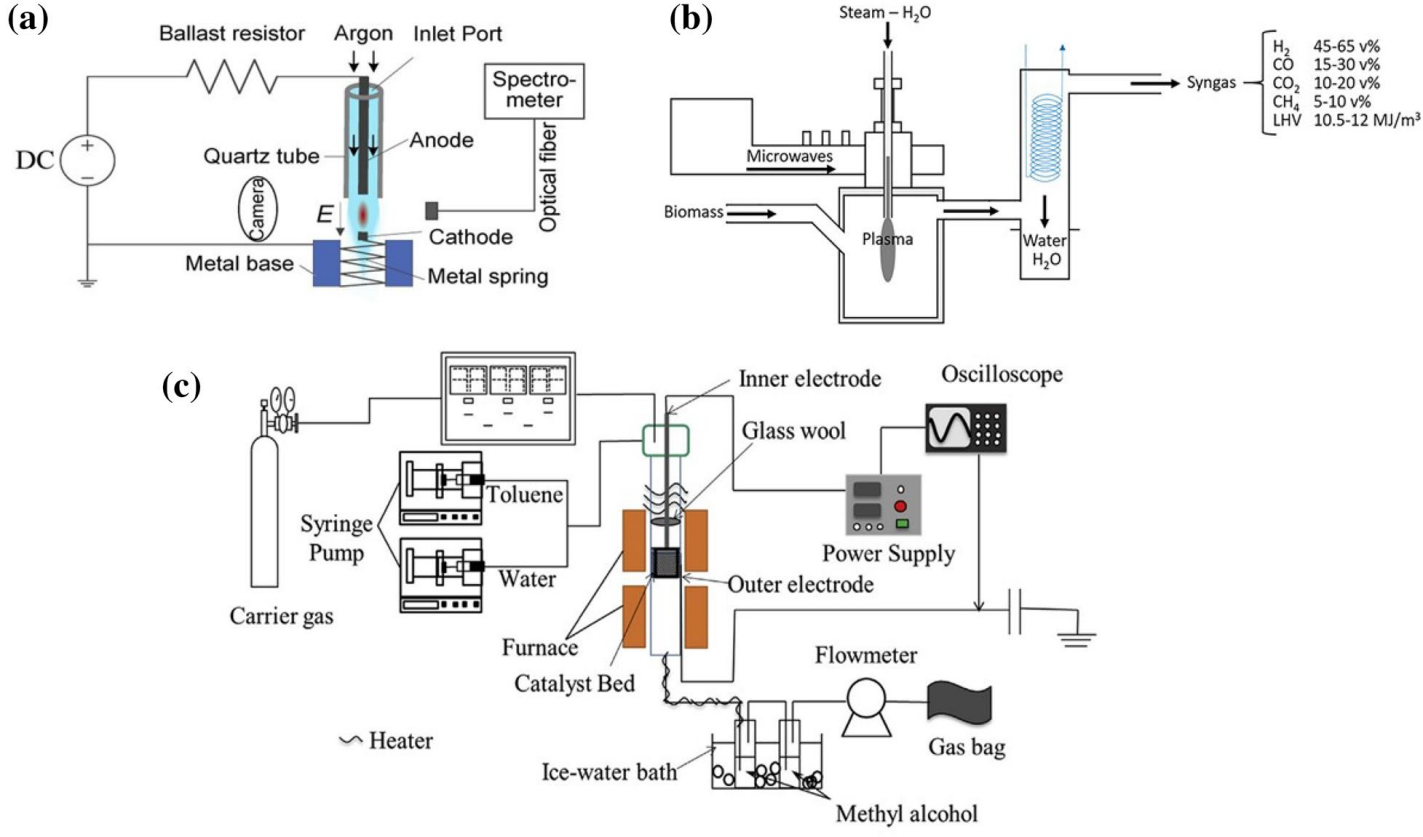
Schematic illustration of **a** GD [29], **b** MD [30], and **c** DBD reactor design for possible biomass conversion application in literature [31]

To produce value-added chemicals from biomass with high efficiency and low power consumption, the plasma reactor and its operating condition have been optimized over past decades. Various type of low-temperature plasma, including glow discharge (GD), microwave discharge (MD), dielectric barrier discharge (DBD), radio-frequency discharge (RFD), and corona discharge (CD), are primarily investigated based on their different mechanisms, applied pressures, and electrode geometries [29–34]. Low-pressure glow discharge (LP-GD) is commonly carried out in a gas atmosphere under low pressure over a small area. The electrons are accelerated easily but the collisions of other heavy ions and neutrals between electrons do not occur frequently. Thus, although the electrons temperature is high, the gas environment can maintain at low temperature under this non- equilibrium state [35]. It enables LP-GD with low-temperature, high energy density, and homogeneous distribution of active species. In the low-temperature MD, microwaves are introduced into the discharge tube or chamber with the assistance of a waveguide or an antenna. The collisions of the electrons and molecules in the reaction gas induce the ionization, dissociation, and excitation [36]. Thus, MD is advantageous owing to its stable discharge, high plasma density, and highly efficient ionization. DBD is a unique gas discharge in which an insulating medium is inserted into the discharge space. The composition of two surface in DBD electrodes benefits to a uniform distribution of electric field and electron density [37–39]. The DBD discharge generates many micro-discharges between the reaction materials with a short service life. Three typical reactor types (GD, MD, and DBD) are shown in Fig. 1a–c, respectively. These technologies exhibit specific properties and advantages [30, 39, 40]. For example, GD can enable a material surface with homogeneous alternation and excellent oxidation. MD presents advantages in terms of its high energy efficiency for producing materials with various properties at high speeds. The DBD was reported with wide applicability since it can be operated under at both atmospheric or the sub-atmospheric pressure. Thus, motivated by recent developments in plasma engineering and science, many attempts have been made to achieve low-temperature plasma-driven biomass conversions [41].

## Application of low-temperature plasma on biomass energy

### Application of low-temperature plasma to biomass pretreatment

The production of value-added biofuels through integrated biorefinery processes requires pre-treatment to enhance the purity of the targeted chemicals. The development of the pretreatment process should be effective for a wide range and high loading of lignocellulosic materials for consideration of industrial application. In general, pretreatment can be principally classified into (i) physical pretreatment, (ii) chemical pretreatment, (iii) bio pretreatment, and (vi) integrated technological pretreatment [40, 42, 43]. The physical pretreatment commonly includes the mechanical milling, extrusion, microwave, ultrasound and so on. Chemical pretreatment typically requires acidic, alkaline, and organic solutions as the processing solvents to loosen the structure and promote facile delignification [44–46]. Bio pretreatment involves the use of enzymes that actively degrade lignin, hemicellulose, and cellulose [43, 47]. However, the rate of biological pretreatment is too slow for commercialization. The integrated technological pretreatment generally involves at least two kinds of basic method to improve the pretreatment efficiency, such as the combination of milling-process with typical alkaline delignification of lignocellulose.

**Fig. 2:**
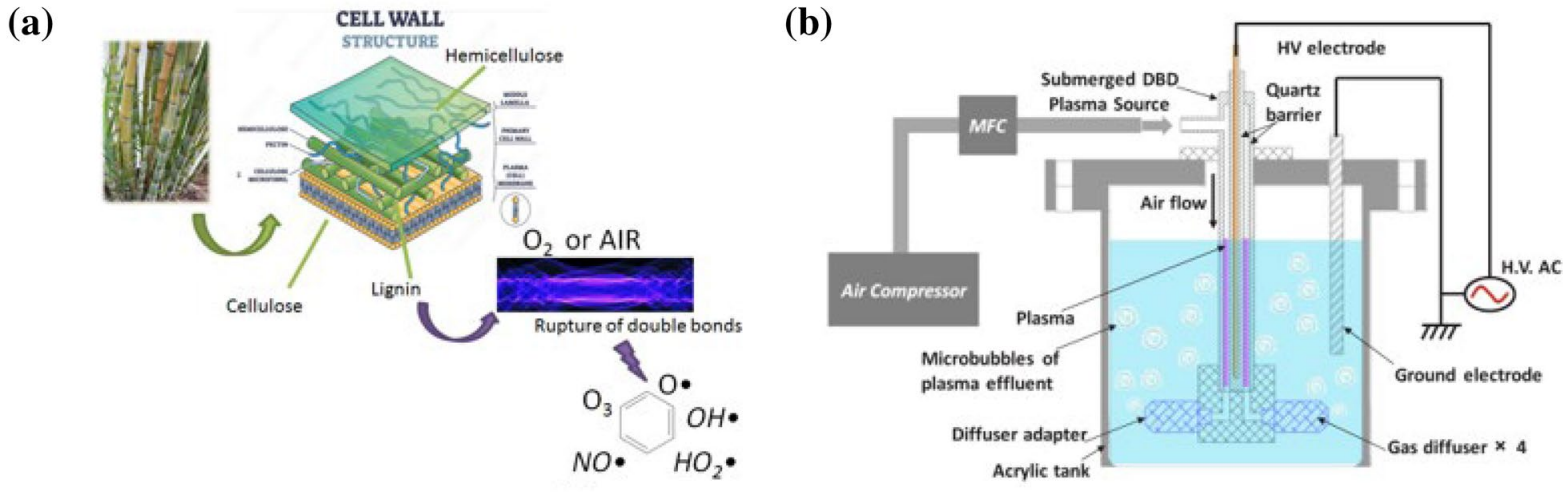
**a** Possible degradation mechanism of lignocellulosic biomass using low-temperature plasma [51]. **b** Schematic representation of submerged DBD plasma for brewer spent grain pretreatment [52]

Low-temperature plasma processing has been studied for technological pretreatment of biomass owing to its relatively short treatment duration, less pollution, and easy recovery of chemicals. In addition, waste treatment and fermentation inhibitors can be simplified in this process [48, 49]. The growth of microorganism can be greatly reduced since the low temperature environment. In the plasma ozonolysis of biomass, O_3_ act as the highly active species in the atmospheric plasma at room temperature [50]. The reactive plasma species in cold gas plasma including hydroxyl radicals (⋅OH), hydrogen peroxide (H_2_O_2_), nitric oxide (NO), and ozone (O_3_) does not only affect the network of cellulose, hemicellulose, and lignin, but also inhibiting the yield of other organic microorganism due to its accompanied disinfection effect by UV, ⋅OH and O_3_, as shown in Fig. 2a. For instance, Muazzam et al. reported the ozonolysis of lignin materials based on plasma technology in DBD microbubble reactor to produce value-added chemicals [50]. The reaction could be controlled from the aspects of reaction time, loading amount and input energy. Optimization of the reaction was performed from the aspects of reaction time, lignin concentration and pH of solvent medium and so on. As a result, the lignin degradation occurs in 1 M NaOH with a processing time of 10 min, lignin concentration of 0.6%, processing temperature between 20 and 25 ℃ was optimized, it shows the vanillin and apocynin with 64.63% and 12.93% of reactive percentage. In the air plasma or oxygen plasma, ozonolysis is a kind of main mechanism in plasma induced reaction. The methoxyl and hydroxyl groups attached on the aromatic rings in the phenolic polymer attracted by ozone with oxidative effect, furthering lead to the breakdown of C-C, C–H, and C–O bonds of lignin structure [51, 52].

Another advantage of low-temperature plasma is that it enables the potential upscale of biomass pretreatment since its high loading amount, low-temperature operation, and absence of inhibitory intermediates. The smaller molecular by-products could be easily recovered by post washing the treated feedstock with water. In summary, the low-temperature plasma can serve as high-performance biomass pretreatment technology on the (i) size reduction of feedstock, (ii) by-products inhibition and (iii) easy post-separation of product from treated feedstock [49, 51]. Further, high loading of feedstock in plasma system also greatly promotes its processing efficiency.

Researchers had made efforts to tailor low-temperature plasma system for extracting lignin to improve the release of fermentable sugars from biomass. As exhibited in Fig. 2b, the submerged DBD plasma was explored to the pretreatment of brewer’s spent grains for sugars production [52]. The process was performed under the voltage of 22–28 kV by using the solvent of acid, alkali, and water for 5–15 min. It also combines the air injection to introduce bubble for improved active species and mass transfer. The result shows an increase of 2.14-fold in yield of the reducing sugar in the following hydrolysis in water media for 10 min. As reported in the literature, the DBD plasma as cellulose pretreatment process enhanced the levoglucosan (LG) yield through pyrolysis within 1 min treatment under ambient air or argon atmosphere [53]. Free radical oriented from the plasma was identified based on the electron paramagnetic resonance (EPR) spectrum. When the highly energetic free electrons collide with cellulose molecules, then the molecules could reach an excited state to easily overcome the barrier for homolytic cleavage. As a result, the levoglucosan yield was enhanced from 58.2 to 78.6% through the integrated plasma-pyrolysis process. This change also allowed the plasma-pretreated cellulose to produce higher yields of LG using lower pyrolysis temperatures. This research suggests that low-temperature plasma could play well in pre-processing of lignocellulosic biomass to achieve the yield of specific product.

### Application of low-temperature plasma to biomass gasification

The gaseous fuel (syngas) derived from lignocellulosic biomass is considered as a green and renewable energy to replace fossil fuel. In recent years, plasma technology is one of most promising technology to be applied in biomass gasification, which is advantageous to the traditional thermal system in processing efficiency, operation, and input cost. However, commercial-scale applications are still inaccessible due to the production of tars with great amount in these processes, it caused the problem of post-processing, and the catalyst deactivation [54]. It is also the reason of the low selectivity of product from biomass by using this method. The abundant impurities also make the post purification to be highly difficult in the biomass gasification system.

**Fig. 3:**
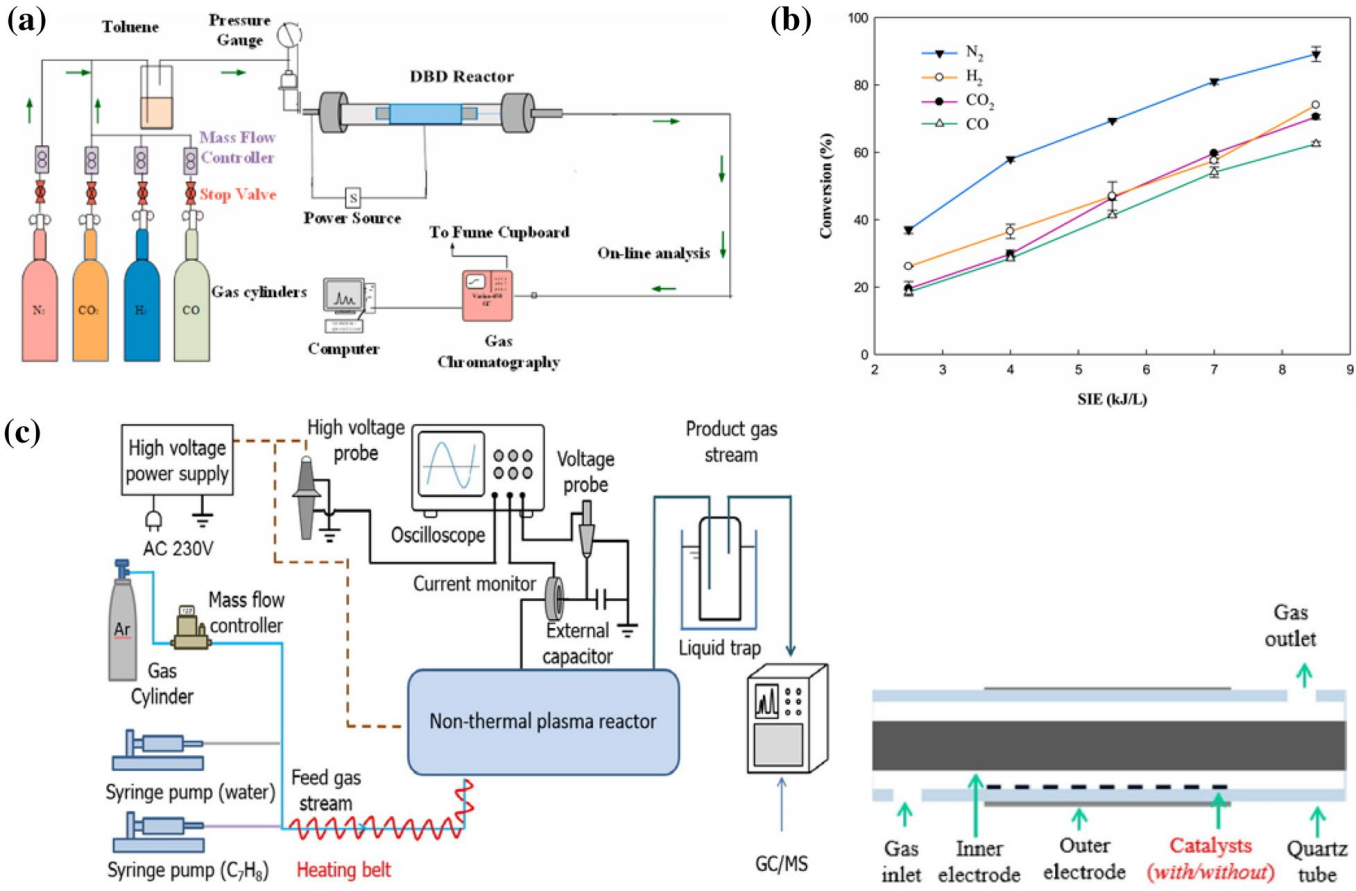
**a** Schematic illustration of biomass gasification using DBD plasma. **b** Effect of carrier gas on biomass gasification performance [55]. **c** Schematic diagram of the DBD plasma-discharge setup and DBD reactor design [62]

In current studies, low-temperature plasma processing of biomass could efficiently solve this problem based on the reactions created by highly reactive species under mild temperature condition. Figure 3a shows a typical experimental setup for lignocellulosic biomass gasification using low-temperature plasma. The plasma reaction system for biomass gasification is composed of four main components, including the DBD reactor, gas cylinder, gas chromatography, and analysis computer. The input power was controlled in the range of 5–17 W. And the gas flow rate was set as 120 ml per min by using a mass flow controller [55]. In the plasma-induced gasification, the distribution of products highly depends on the property of carrier gas. In general, CO, CO_2_, H_2_, or N_2_ is used as the carrier gas. N_2_ has been shown to give the maximum conversion among all carrier gases. The performance of plasma application on toluene decomposition in different carrier gases was investigated following the order CO < CO_2_ < H_2_ < N_2_, as shown in Fig. 3b. The tar removal of toluene achieved the maximum result of 89.1% in N_2_ atmosphere at the specific input energy (SIE) of 8.5 kJ/L and 1.43 s. In the previous study, the air and N_2_ were investigated with high activity and low input cost as the carrier gas since its high activity, easy ionization, and low cost [34, 56–58].

In recent years, a hybrid process combining chemo-catalysis and plasma technology has drawn increasing research attention in biomass conversion. In terms of chemical conversion of biomass in gas phase or liquid phase, the utilization of heterogeneous catalyst is more encouraged than homogeneous catalyst since its easy separation and recyclable property [59–61]. Thus, the heterogeneous catalyst-assisted plasma technology for biomass conversion has been widely investigated in current days, especially for the biomass gasification. The synergistic effect of catalyst and plasma not only lower the activation energy but also improve the gasification performance via activated chemical species and electrons. It combines the advantages of fast and low-temperature reactions and catalysts-oriented reaction pathway. Figure 3c exhibits the experimental setup and DBD reactor in a hybrid plasma-catalytic system for the tar removing. Compared to the DBD plasma process in absence of catalyst, a catalyst with different active site loading was placed in the plasma region along the bottom of quartz tube. The partial filling of catalyst in the plasma reactor could consequently promoted the plasma-catalytic chemical reaction. The coupling of plasma and a tailored Ni@Al_2_O_3_ catalyst efficiently reduce the coke deposition on the catalyst surface to enhance the sustainability [62]. An optimal toluene conversion efficiency was obtained at 52% over the catalyst of 20 wt% Ni/Al_2_O_3_ in this plasma process.

### Application of low-temperature plasma to biomass liquefaction

The gasification of biomass mainly focuses on the production of gaseous fuel. Although high conversion efficiency has been achieved in past decades, but the application scope is limited. Solvent-based catalytic liquefaction exhibits excellent performance on conversion of biomass into value-added chemicals [63]. In conventional methods, the liquefaction is mainly driven by high temperature and specific solvent to assist the catalyst to break the strong bonding in the network of lignocellulose. The solvents utilized for the biomass liquefaction can be typically separated into alkaline and acidic solvent. The water content is one of the significant factors to the acidic liquefaction because the ionized hydrogen plays an important part in this process [64, 65]. However, the conventional liquefaction process required intensive reaction condition with hazardous chemicals and long reaction time. Therefore, the development of low-temperature plasma process for biomass liquefaction solves this problem by the introducing highly active species during the processing.

**Fig. 4:**
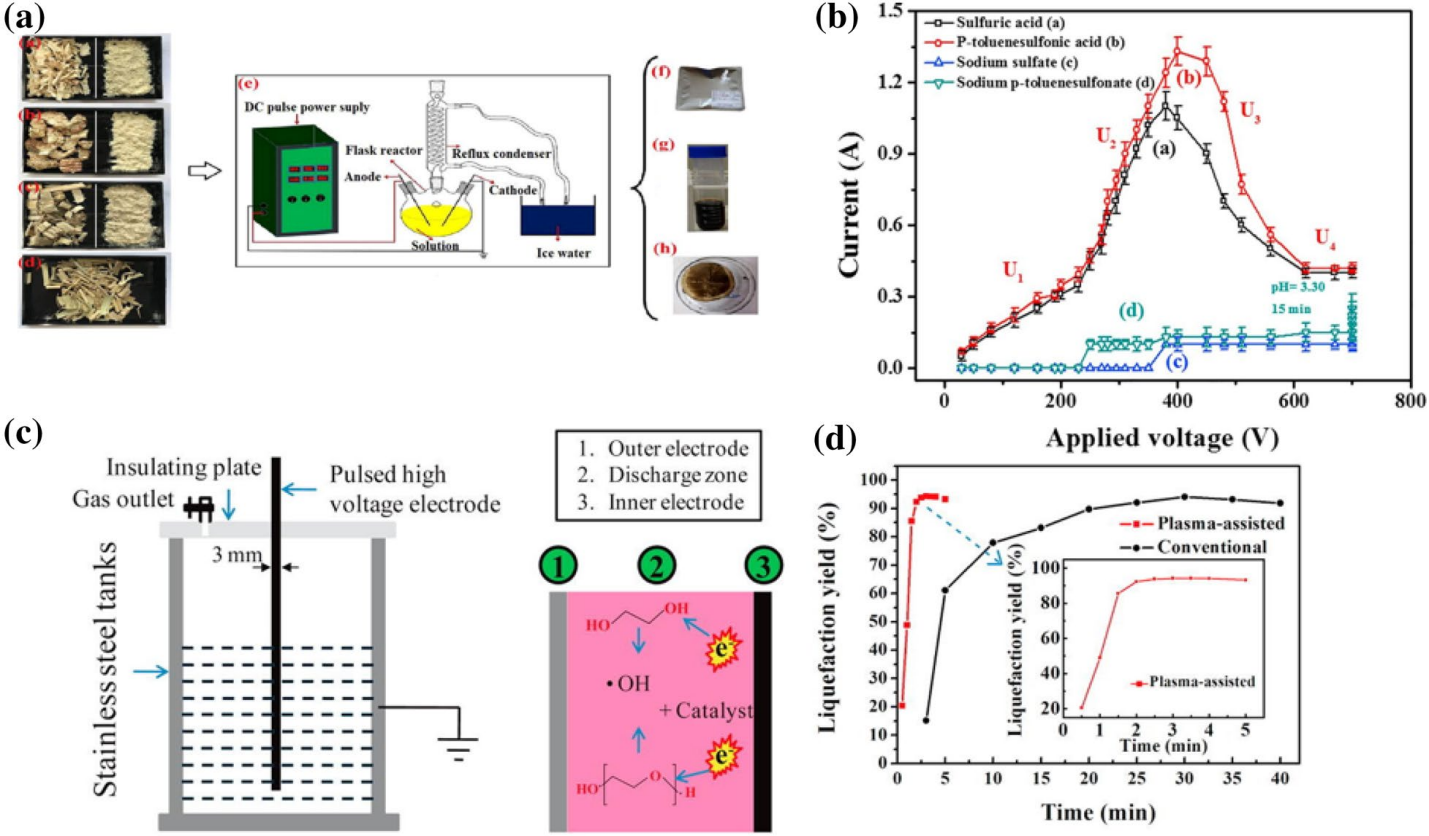
**a** Schematic illustration of biomass liquefaction using plasma technology. **b** The relationship between applied voltage and current in various catalysts [66]. **c** Schematic diagram of the DBD plasma-discharge setup. **d** Comparison between conventional method and plasma-assisted liquefaction [67]

Low-temperature plasma process enhanced the liquefaction performance *via* the plasma-induced reactions, including applying free radicals of ⋅OH, ⋅H and electrons functionalized as active species for bond cleavage [66]. The primary reaction of biomass liquefaction could be driven by the direct collision between the energetic electrons and the molecules of biomass and solvent, which resulted in cleavage and degradation of the cellulose, hemicellulose and lignin and forming the various chemicals, such as phenols, furfurals, and monosaccharides. Assisted by the plasma-induced active species, the liquefaction performance of the catalytic reactions could be significantly improved. Furthermore, the heating in plasma electrolysis plays an essential part in improving liquefaction efficiency. Figure 4a, b depicts plasma technology coupled with a reflux system for the catalytic liquefaction of sawdust, corn cob, corn stalk, and rice straw [66]. The discharge characteristic and the performance of applied catalyst, including sulfuric acid, p-toluene-sulfonic acid, sodium sulfate, and sodium p-toluene sulfate were investigated. Zhou et al. proposed a liquid-phase micro plasma-assisted technology for bamboo-shoot-shell liquefaction in sulfuric acid, where the plasma was generated by a pulsed high-voltage DC power supply [67]. A schematic of this system is shown in Fig. 4c. In addition to the acidic processing of bamboo, active hydroxyl species play a major role in the oxidative decomposition of the lignocellulosic network. Plasma-driven liquefaction achieves approximately ten times the catalytic efficiency of the conventional process, reaching the maximum product yield within 5 min, as shown in Fig. 4d. On the other hand, the liquefaction of biomass in acidic solvents not only corrode the equipment but also affect the final purification of the bio-products. Except the typical acidic catalysts, some catalysts based on sodium salts with released H ions in the system were integrated. Xi et al. utilized industrial sodium salts as catalysts with plasma for biomass liquefaction to improve the liquefaction rate under various water contents and lower energy consumption [66]. Comparative study between sodium sulfate and sodium *p*-toluene sulfonate demonstrates the higher solubility and H^+^ ion benefits to the liquefaction yield. The liquefaction yield of sawdust by using sodium sulfate and sodium *p*-toluene sulfonate as the catalyst are 60.63% and 83.51% after optimization, respectively. The temperature was optimized above 100 ℃ over these salts’ catalyst in plasma electrolysis liquefaction, because the degree of ionization of H determined the catalyst performance. In common, the gas plasma can be operated in a wide range of pressure, thus the composition of the gas source of gas flow rate (pressure) affects the catalysis performance due to the different activity and its interaction with the feedstock. Overall, the parameters such as the S/C ratio (stream/carbon ratio), nature of solvent and gas pressure are crucial to the catalysis performance in the corresponding integrated plasma-catalysis system for biomass gasification and liquefaction. Thus, detail studies of the influence of these parameters should be further conducted for the optimization of the reaction system in near future [68].

### Application of low-temperature plasma to the modification and the synthesis of catalysts

Homogeneous catalysts, in which the active sites are in the same phase as the reactants, typically resulting in high transformation efficiency, and heterogeneous catalysts have a significant advantage in terms of recyclability over homogeneous catalysts [69]. For instance, sulfonated carbon as a kind of typical solid acid catalyst has been applied into various biomass transformation processes, such as the cellulose hydrolysis, fatty acid esterification, ketalization reaction and so on [70]. However, most of sulfonated catalysts fabricated from conventional process still confined to the use of hazardous sulfonation chemicals, including high concentration of sulfuric acid, fuming sulfuric acid, chlorosulfonic acid, as well as harsh reaction condition with elevated temperature and high pressure. Hence, it is highly desirable to design a green synthesis route to replace the conventional process. Recently, low-temperature plasma technology has rapidly emerging as an efficient and soft preparation approach for heterogeneous catalyst. Plasma engineering was reported that it can synthesize carbon or metal-carbon hybrid nano materials by selecting different precursors and controlled with different functional groups and active site doping [71–73]. The process provides great potential for the synthesis and modification of carbon acid catalysts for biomass upgrading.

**Fig. 5:**
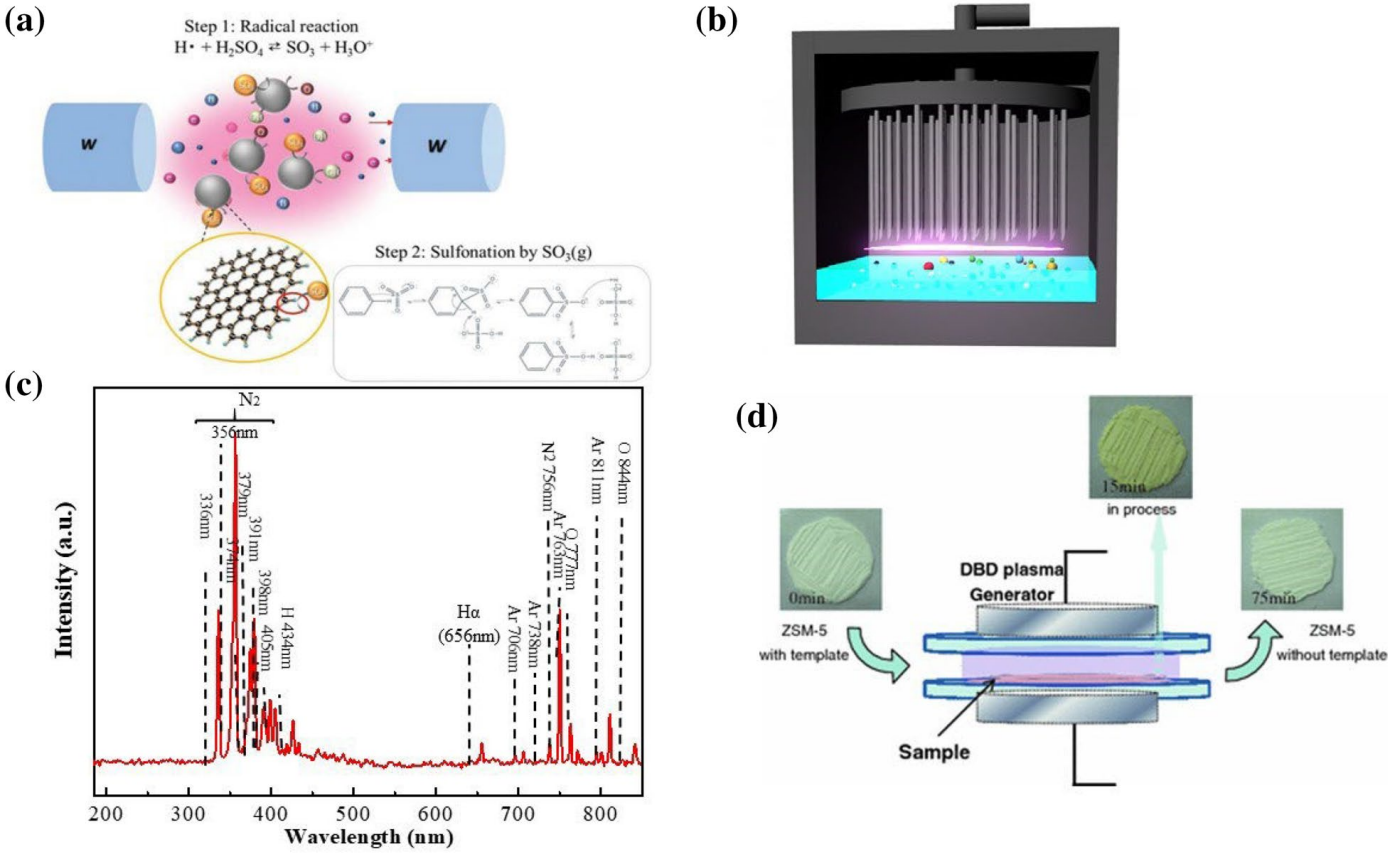
**a** Schematic illustration of solution plasma sulfonation [74]. **b** Gas–liquid interfacial plasma for carbon sulfonation. **c** OES spectrum of gas–liquid interfacial plasma in air/Ar atmosphere [75]. **d** DBD plasma for zeolite preparation [76]

Li et al. has reported various low-temperature plasma processes for various solid acid synthesis [74]. First, plasma engineering was investigated to introduce acidic functional groups on the surface of carbon black under dilute sulfuric acid of 1 mol/L. As the schematic illustration of the mechanism in Fig. 5a, the activation of sulfate ion by the H radicals firstly induce the generation of SO_3_ species, followed by the surface attachment of –SO_3_H groups on the carbon network. Except the sulfonic acid groups, other oxygenated groups including hydroxyl and carboxyl simultaneously anchored on the carbon surface based on the massive amount of H and OH radicals generated in the plasma system. The texture property of chemical structure of the original and plasma-treated carbon black have been characterized by using Brunauer Emmett Teller method (BET) and infrared spectroscopy (IR). Slight increases of specific surface area and pore size were observed after plasma engineering, where the corresponding signal of C–O, O–H, and S=O adsorption confirmed that the anchoring of acidic groups on the carbon network. The resulting cellulose conversion rate of plasma-sulfonated carbon black (40.1%) is around four times higher than that of the commercial catalyst (< 10%) in the comparison test under identical hydrolysis condition [74]. Another type of plasma engineering, named gas–liquid interfacial plasma (GLIP), has also been explored as a novel green sulfonation process. GLIP were performed under the air/Ar-dilute acid system to graft acid groups on various carbon substrate around room temperature [75]. The active species generated in the plasma was confirmed from the OES spectrum as shown in Fig. 5b. The major peak belongs to H_α_ located around 653 nm where N_2_-related radicals can be found around 330–400 nm. Accordingly, the plasma chemistry of gas–liquid interfacial plasma sulfonation process can be classified into three phases composed of (i) gas phase, (ii) gas–liquid interface, and (iii) liquid phase, as exhibited in Fig. 5c. These radicals can be transported into the liquid phase to induce sulfuric acid dissociation and further proceed to sulfonation with SO_3_ radicals on the carbon surface. The structural property after GLIP treatment demonstrated more defect was generated on carbon surface after anchoring the acidic functional groups. In addition, the anchoring of C–O, O–H, and S=O confirms was clearly presented in surface analysis. Comparatively, the gas–liquid plasma sulfonation process shows high processing efficiency and larger amount of weak acid groups grafting than conventional hydrothermal sulfonation. In particular, GLIP-sulfonated carbon acid catalysts presented extremely high catalytic stability of 96.6–98.2%. Therefore, plasma engineering not only improve the catalysis stability but also reduce the use of corrosive chemicals, which is beneficial for sustainable utilization.

Not only limited to surface modification or materials synthesis, low-temperature plasma can be applied as the post-treatment of catalysis synthesis. As shown in Fig. 5d, DBD plasma is an effective process for template removal during zeolite preparation [76]. During the plasma-induce template removing, the average voltage and average power were 14 kV and 250 W, respectively. The external heating or cooling system to assist the plasma engineering in this system is not required. Compared to traditional calcination method, DBD plasma operated at comparatively lower temperature (125 °C) with a removal speed improved to eight times. Comparatively, the DBD based template removal of ZSM-5 zeolite made the structure changes of less dramatic than that using thermal calcination. In addition, the texture of the catalyst treated using the low-temperature plasma method can be controlled.

## Conclusion

In summary, the application of low-temperature plasma technology to biomass conversion is discussed in this review, along with pretreatment of lignocellulosic biomass, gasification and liquefaction of biomass, and the preparation of heterogeneous catalysts for chemo-catalytic biomass conversion. Due to the great structural recalcitrance of lignocellulosic biomass materials, an effective pretreatment should be implemented to improve the downstream upgrading. Biomass gasification and liquefaction are efficiently promoted by introducing trapped active species in the lignocellulose network. Since a large number of highly energetic electrons and free radicals are generated from the plasma, these active species can effectively promote mass transfer and the interaction between the reactants and catalyst, therefore reducing the activation energy and accelerate the chemical reaction rates. As a result, integrating low-temperature plasma with catalyst could further improve the performance of biomass conversion.

Additionally, it is feasible to target specific products with high selectivity by carefully tailoring the plasma-catalyst system. Due to the mild operating environment, low-temperature plasma engineering shows limited damages during the surface functionalization of heterogeneous catalyst, which ensured high recycle utilization. For the synthesis of solid catalysts, plasma engineering contributes to improving synthesis efficiency, mesoporous structure and surface area compared to other conventional processes. Plasma engineering benefits to the well distribution of active species in the support materials. Despite the above benefits, low-temperature plasma technologies are still at a developing stage due to its high energy consumption. First, plasma-assisted technology has undergone great progress in reactor design, making it accessible on an industrial scale yet the cost of plasma generator is still relatively high. For instance, the nano-pulse power generator multiplies the number of active species compared with using a traditional power supply. Combining the effective sequence of pretreatment and post-processing could highly improve the working efficiency. Secondly, the understanding of plasma chemistry and the mechanism of plasma- catalytic reactions are still under investigation for the development and optimization of plasma technology in the field of biomass transformation. For example, the active species during processing in various kinds of media can be potentially detected and analyzed through advanced in situ chromatography, which is vital to further broaden the application of low-temperature plasma technologies in biorefinery. Along with these emerging studies related to improving the energy efficiency and understanding the detail plasma-related mechanisms, we believe that low-temperature plasma is a highly feasible alternative to other traditional methods for biomass conversion.

## Data Availability

Not applicable.
